# Supplementary figures and images for: Functional correlates of cognitive performance and working memory in temporal lobe epilepsy: Insights from task-based and resting-state fMRI
Source: PLoS One. 2024 Mar 13;19(3):e0295142. doi: 10.1371/journal.pone.0295142 (PMC10936866; doi:10.1371/journal.pone.0295142)

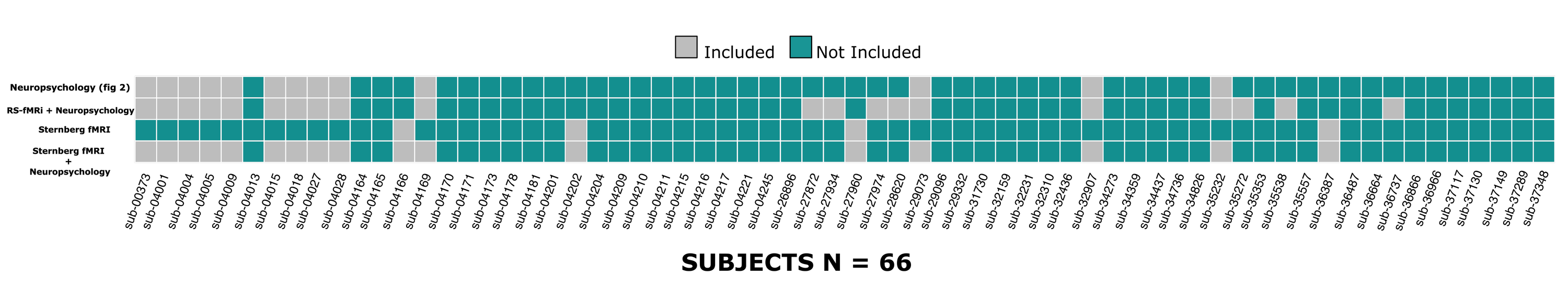

Supplement: S1 Fig — (TIF) [file pone.0295142.s001.tif]

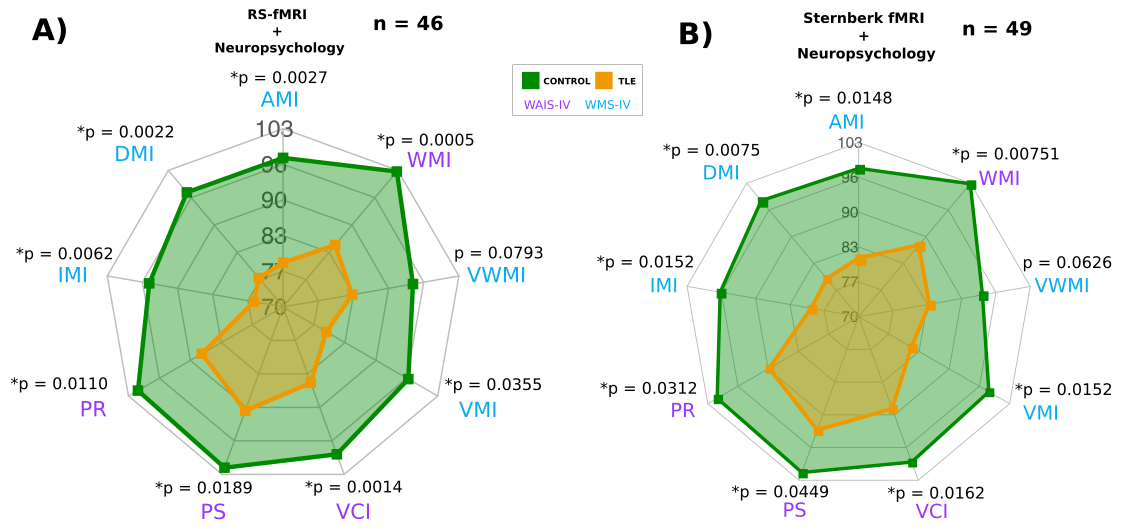

Supplement: S2 Fig — (TIF) [file pone.0295142.s002.tif]

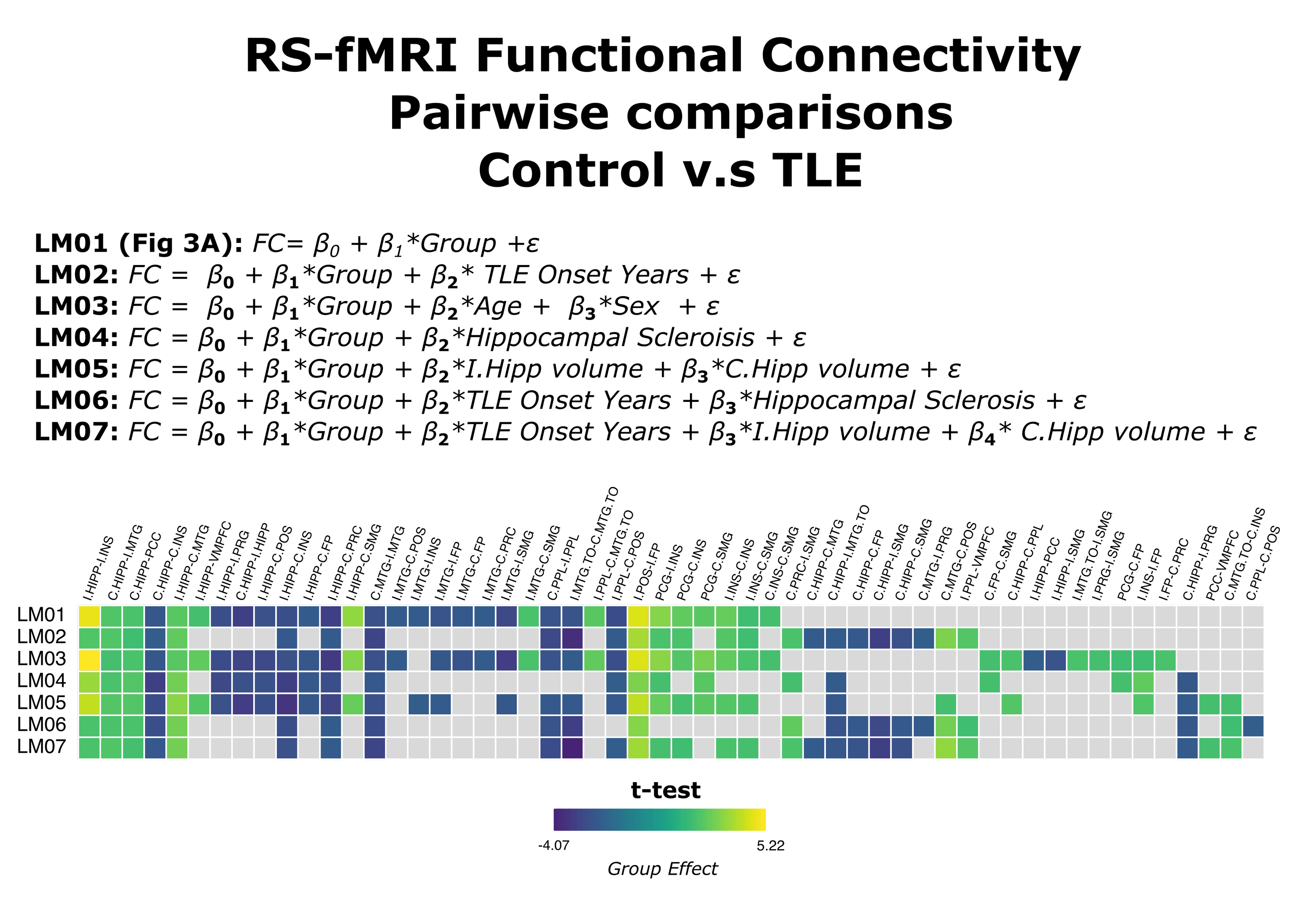

Supplement: S3 Fig — p-value < 0.05, uncorrected. (TIF) [file pone.0295142.s003.tif]

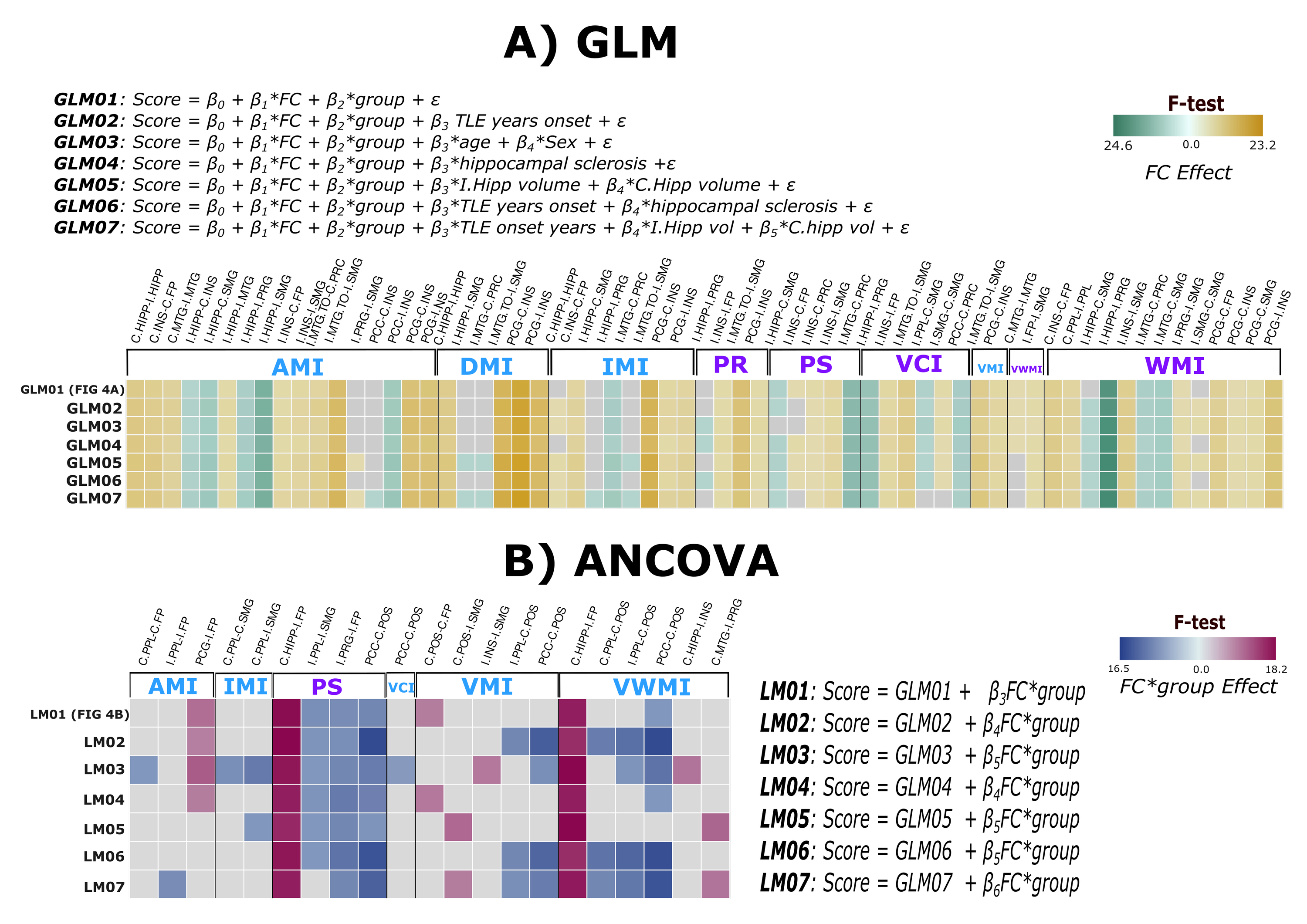

Supplement: S4 Fig — C: Scatterplots of GLM significant associations (n = 46). D: Scatterplots of ANCOVA significant associations (n = 46). E: Associations between Cognitive scores and Functional connectivity adjusted for covariates. (ZIP) [file pone.0295142.s004.zip › S4E_Fig.tif]

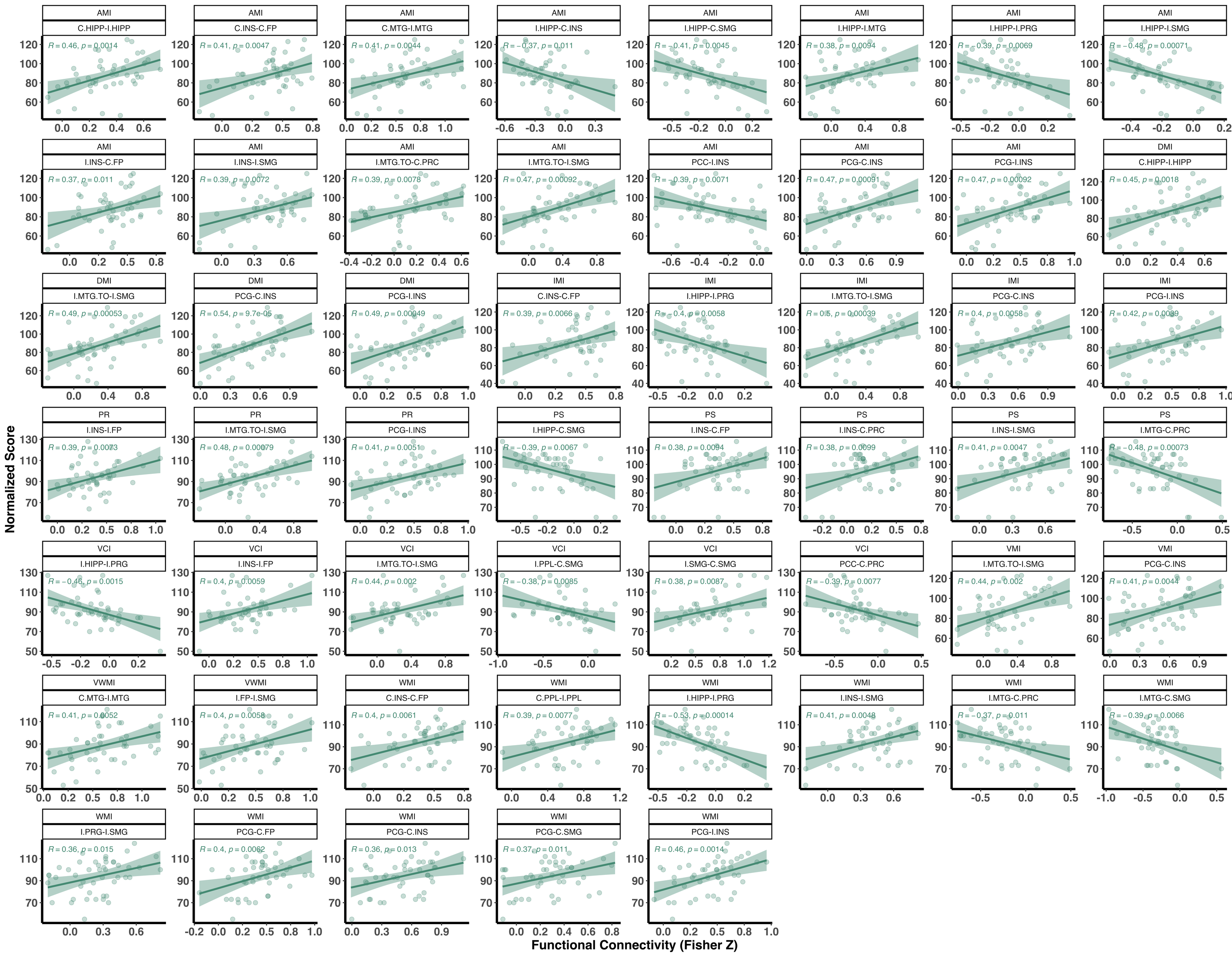

Supplement: S4 Fig — C: Scatterplots of GLM significant associations (n = 46). D: Scatterplots of ANCOVA significant associations (n = 46). E: Associations between Cognitive scores and Functional connectivity adjusted for covariates. (ZIP) [file pone.0295142.s004.zip › S4C_Fig.tif]

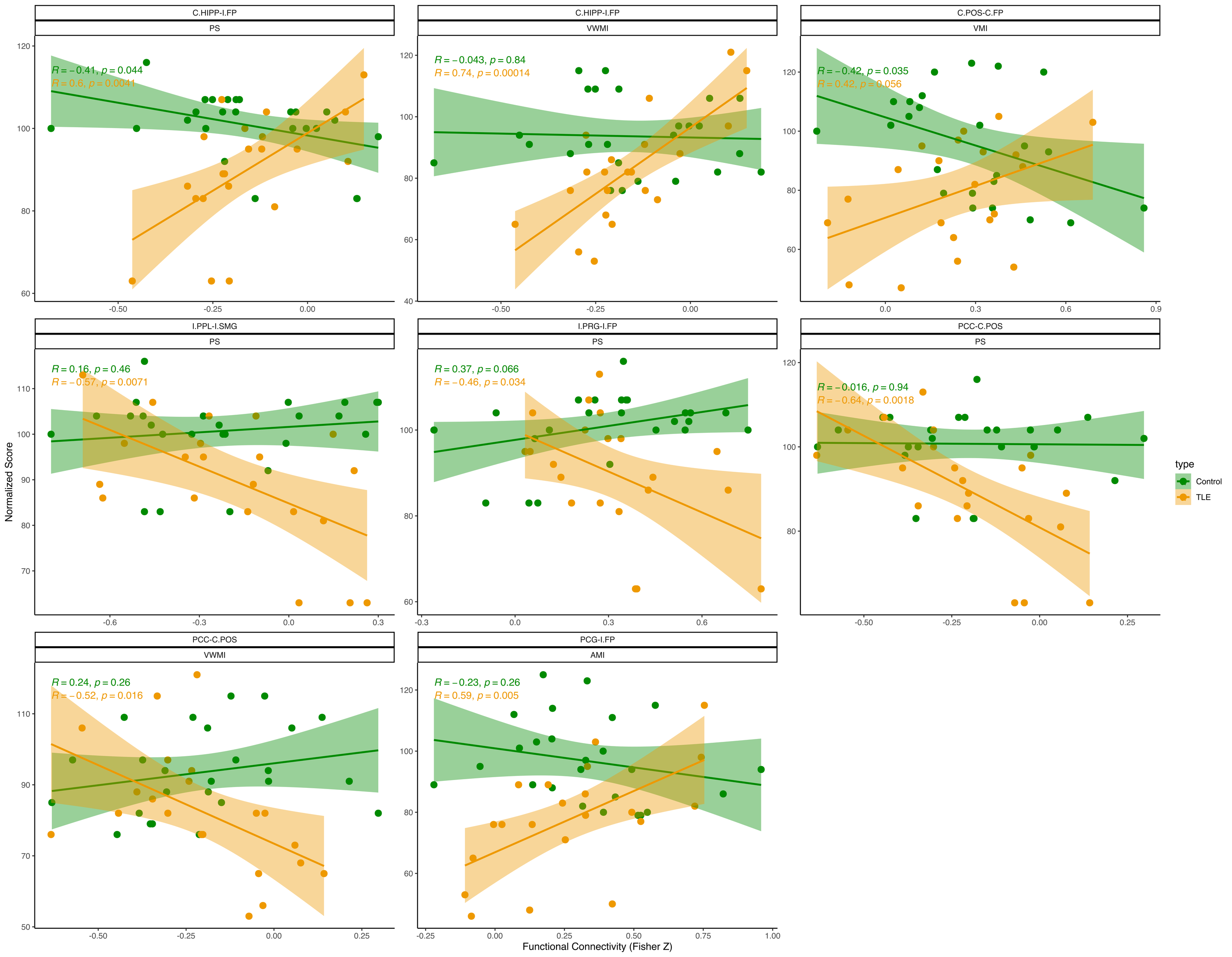

Supplement: S4 Fig — C: Scatterplots of GLM significant associations (n = 46). D: Scatterplots of ANCOVA significant associations (n = 46). E: Associations between Cognitive scores and Functional connectivity adjusted for covariates. (ZIP) [file pone.0295142.s004.zip › S4D_Fig.tif]

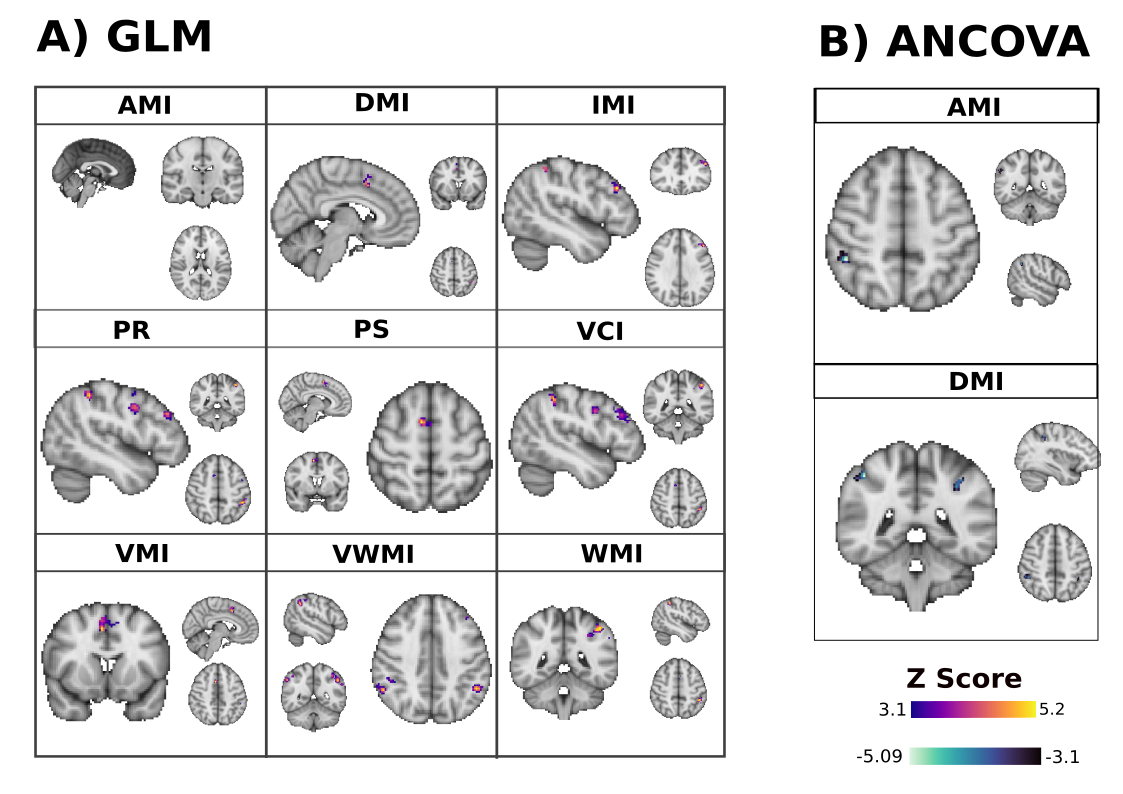

Supplement: S5 Fig — Voxel-wise results for GLM (A) and ANCOVA (B) for all cognitive scores. Interactive maps are available at https://github.com/alffajardo/TLE2023_fMRI. (TIF) [file pone.0295142.s005.tif]
