# Supplementary material for: Functional correlates of cognitive performance and working memory in temporal lobe epilepsy: Insights from task-based and resting-state fMRI
Source: PLoS One. 2024 Mar 13;19(3):e0295142. doi: 10.1371/journal.pone.0295142 (PMC10936866; doi:10.1371/journal.pone.0295142)
Supplement: Attachment — Submitted filename: response.docx [file pone.0295142.s007.docx]

**Functional Correlates of cognitive performance and working memory in temporal lobe epilepsy: Insights from task-based and resting-state fMRI.**

**Response to Reviewers**

We thank the two Reviewers for their overall positive feedback and appreciation of our work.

Reviewer #1 suggested the additon of information to the public data set, namely medication used by patients, and age of epilepsy onset. The new version of the data set now includes this information.

Reviewer #2 provided us with the following suggestions, which we have addressed in this version:

**1.) The additional analyses investigating whether clinical factors like age at onset of seizures, age at time of study, birth sex and presence of HS impact the observed effects should be elaborated on in the Discussion.**

We have included this in our Discussion (page 23), reproduced here:

When accounting for demographic and clinical variables, the majority of findings were reproduced with a similar magnitude and direction (S3 fig). A reduction in the effect was observed when adjusting for duration of illness and structural integrity of hippocampi (volume and presence of hippocampal sclerosis). Thus, these variables are an important source of variance that account for the observed  FC alterations in TLE patients. This effect was particularly noticeable in edges that involved ipsilateral hippocampus and ipsilateral medial temporal lobe nodes. Interestingly, adjusting for these covariates also revealed alterations in contralateral hippocampus-TPN edges. These alterations, as well as decreases of DMN-TPN anticorrelations may account for the cognitive deficits observed in the TLE group.

**2.) S2 Fig: The authors state that S2 Fig shows similar/ nearly identical results to Figure 2, however, no statistical results for S2 Fig are presented in the Figure or text/caption.**

Statistical results were added to S2 Figure, as suggested.
